# Building Capacity for Rigorous Health Research Through Grant Writing Coaching

**DOI:** 10.3390/ijerph23050668

**Published:** 2026-05-19

**Authors:** Yulia A. Levites Strekalova, Rachel Liu-Galvin, Stacey Gorniak, Hongmei Wang, Felicite Noubissi, Adriana Baez Bermejo, Jonathan Stiles, Mohamed Mubasher, Elizabeth Ofili

**Affiliations:** 1 Department of Health Services Research, Management, and Policy, College of Public Health and Health Professions, University of Florida, Gainesville, FL 32611, USA; 2 Department of Health and Human Performance, College of Liberal Arts and Social Science, University of Houston, Houston, TX 77204, USA; 3 Department of Pharmacy Practice and Administration, College of Pharmacy and Health Sciences, Texas Southern University, Houston, TX 77004, USA; 4 Department of Biology, Jackson State University, Jackson, MS 39202, USA; 5 Department of Otolaryngology–Head and Neck Surgery, School of Medicine, University of Puerto Rico, San Juan, PR 365067, USA; 6 National Research Mentoring Network (NRMN) and RCMI Coordinating Center, Morehouse School of Medicine, Atlanta, GA 30310, USA

**Keywords:** grant writing coaching, excellence in biomedical research, early-stage investigators, research capacity building, realist evaluation

## Abstract

**Highlights:**

**Public health relevance—How does this work relate to a public health issue?**

- There is a critical need to strengthen research mentoring for underrepresented groups in medical science to advance equity in the health research workforce and build sustainable research capacity.
- Limited access to grant-writing training and mentorship remains a key barrier to funding success among early-stage investigators, particularly those from underrepresented backgrounds.

**Public health significance—Why is this work of significance to public health?**

- This study evaluates a mentorship and grant-writing program designed to support the professional development of early-stage investigators, including those from underrepresented groups, with a focus on developing grant-writing skills. Using a realist evaluation framework, the findings identify the context, mechanisms, and outcomes contributing to the success and challenges of the program.

**Public health implications—What are the key implications or messages for practitioners, policy makers and/or researchers in public health?**

- Investment in mentorship and structured grant-writing support may enhance research capacity and contribute to a more diverse, equitable, and sustainable public health workforce.
- Insights from this study can inform the design, implementation, and evaluation of scalable programs to support early-career investigators, including those from underrepresented groups, in developing grant-writing skills and producing competitive proposals.

**Abstract:**

Background: The National Research Mentoring Network (NRMN) aims to enhance capacity in the biomedical research workforce through mentorship, professional development, and networking. This study focuses on the Strategic Empowerment Tailored for Health Equity Investigators (NRMN-SETH) program, which supports early-stage investigators (ESIs), including those from underrepresented groups, in developing grant-writing skills. Using a realist evaluation framework, this study explores the contexts, mechanisms, and outcomes contributing to the program’s effectiveness. Methods: A directed content analysis approach was employed, guided by the realist evaluation framework. Data sources included speaker slides, moderator notes, and participant observations from the 2024 RCMI NRMN-SETH session. Context-mechanism-outcome configurations were analyzed to identify key stakeholders, enabling factors, and barriers to success. Results: Five key mechanisms emerged: social support, peer accountability, knowledge of grant-writing strategies, technical grant-writing knowledge, and access to mentoring. Critical contexts included protected time for grant writing, access to subject-matter experts, and participant readiness. Institutional leadership support and cross-institutional collaborations were identified as essential for sustainability. Conclusions: The NRMN-SETH program effectively supports ESIs through mentorship and technical guidance, fostering equitable participation in biomedical research. Future efforts should focus on institutional investment in mentorship, grant readiness, and expanded access to subject-matter experts to enhance the program’s scalability and long-term impact.

## 1. Introduction

### 1.1. Gaps and Efforts in Workforce Research Capacity Development

Addressing disparities in the health research workforce and building capacity in underserved communities are essential for rigorous and impactful biomedical research [1]. The National Institutes of Health (NIH) Diversity Program Consortium (DPC), encompassing initiatives like the National Research Mentoring Network (NRMN) and Building Infrastructure Leading to Diversity (BUILD), has created inclusive environments through mentoring and capacity building at low-resourced and minority-serving institutions (MSIs) [2]. These programs tailor training to underrepresented groups, employ equity-promoting frameworks, and foster community sustainability and networked expertise.

The Research Centers in Minority Institutions (RCMI) program strengthens institutional infrastructure and facilitates collaborative partnerships with research-intensive institutions. These partnerships leverage bidirectional learning and shared resources to reduce disparities in grant competitiveness and scientific output [3]. Mentorship emerges as a cornerstone of success, with targeted training programs based on the format of the Entering Mentoring and Mentoring Up for Early Career Investigators curricula, addressing the specific needs of early-stage investigators [4,5]. These initiatives emphasize skills such as effective communication and achieving independence, thus contributing to long-term career progression [4,6,7].

Virtual environments, such as the Health Equity Learning Collaboratory, further bridge gaps in mentorship and peer support by connecting investigators across diverse geographies, offering critical resources for grant preparation, and accelerating proposal submissions [8]. Systematic reviews underscore the need for sustainable funding models and robust outcome evaluations [9,10]. Collectively, these programs demonstrate the value of integrated mentorship and institutional collaboration in building an inclusive, sustainable biomedical research ecosystem.

### 1.2. The NRMN-SETH Approach

Grant-writing skills drive research capacity and independence in a research career, which has been recognized and supported by the NRMN. The NRMN is a federally funded initiative aimed at enhancing representation in the biomedical research workforce through culturally responsive mentorship, networking, and professional development [11]. Established as part of the NIH’s Diversity Program Consortium (DPC) [1], the NRMN addresses the critical need for improving research mentoring and career preparation for underrepresented groups in biomedical sciences [12]. The NRMN’s approach to workforce development is multifaceted, focusing on matching, based on background discipline, mentees across career stages with mentors and coaches, training participants in effective mentoring relationships, and providing resources for professional development [11]. The network has developed virtual communities of practice like MyNRMN, a national mentoring and networking platform designed to enhance connectivity and rigor in biomedical sciences, further supporting this mission [11].

A key component of NRMN’s strategy is its emphasis on developing grant-writing skills for early-stage investigators (ESIs), particularly those from underrepresented groups and MSIs [13]. The NRMN-SETH (Strategic Empowerment Tailored for Health Equity Investigators) program, for instance, evaluates the impact of interventions addressing the developmental networks of diverse ESIs [14,15]. This approach includes regular pre-application mock NIH-like study sections to advise ESIs with completed applications, providing valuable feedback, and improving their chances of securing funding [14]. The NRMN’s efforts extend beyond individual mentoring to institutional capacity building. For example, the RCMI program collaborates with the NRMN to leverage inter-RCMI Center shared expertise and resources, supporting synergistic participation in national research networks [3,15]. This approach helps address disparities in resource access between research-intensive majority institutions and MSIs, potentially leveling the playing field in grant proposal competitiveness [3].

The NRMN’s impact is evident in its reach and outcomes. Within two years of its inception, over 2000 mentors participated in NRMN mentor training, and 2500 mentees (75% from underrepresented groups) engaged with the network [12]. The network has also supported numerous ESIs in grant writing, with 225 participants receiving intensive grantsmanship coaching [12]. From an organizational perspective, NRMN demonstrates the power of collaborative networks in addressing complex societal challenges. Its approach aligns with theories of social capital and network effects, leveraging diverse expertise and resources across institutions to create a more inclusive research ecosystem [16,17]. However, challenges remain in achieving long-term, sustained growth in biomedical workforce research capacity. As noted by Vishwanatha et al., measurable changes in the workforce’s demographic composition take time and cannot be achieved by a single program alone [12].

The NRMN represents a comprehensive, theory-driven approach to biomedical workforce development. By addressing multiple facets of career development, from mentorship to grant-writing skills, and leveraging institutional partnerships, the NRMN is creating a more diverse and inclusive biomedical research community [18]. While the NRMN approach to grant-writing coaching shows promising results, there remains a critical need to explore replicable contexts and mechanisms to sustain capacity building for RCMI-based ESIs. Future research could benefit from applying realist evaluation frameworks to further understand the contextual factors and mechanisms driving the NRMN’s successes and challenges. Thus, this paper aims to present the evidence and ideas exchanged at the 2024 RCMI NRMN session and to answer the following research question: What grant writing support for research capacity development works, for whom, and in what contexts?

## 2. Methods

### 2.1. Conceptual Framework

Realist evaluation is a theory-driven approach to understanding complex social interventions by identifying which specific components are effective, the populations they benefit, and the conditions under which they succeed [19]. Rooted in critical realism, this framework seeks to unpack the intricate relationships between context, mechanisms, and outcomes (CMO configurations) in program evaluation [20]. At its core, realist evaluation acknowledges that interventions operate within complex social systems and that their effectiveness is contingent upon the interplay of various contextual factors [21]. This approach moves beyond assessing whether an intervention works to exploring how and why it works in specific settings. The concept of mechanisms is central to realist evaluation [22]. Mechanisms are understood as the underlying processes that generate outcomes within the contexts of organizational capacity, mentor availability, and accessibility of scientific space and equipment.

Realist evaluation has gained traction in healthcare and biomedical research, offering a nuanced framework for evaluating complex interventions in these fields [23]. It provides a structured approach to understanding the multifaceted nature of program implementation and outcomes, which is particularly useful in evaluating efforts to diversify the biomedical workforce [18]. However, implementing realist evaluation can be challenging due to its complexity and the need for researcher creativity and reflection [19]. It requires a deep understanding of context and the ability to theorize underlying mechanisms. In biomedical workforce development, realist evaluation offers a promising approach to understanding the nuanced factors that shape research capacity-building initiatives. By examining the interplay of contextual factors, mechanisms, and outcomes, it can provide valuable insights into how to effectively design and implement programs to increase rigor and representation in the biomedical research workforce [18].

### 2.2. Participants and Data Sources

The data sources used in this study included speaker slides and moderator participant-observer notes collected during the RCMI NRMN session. The analysis used slides and moderator notes from the RCMI session conducted on 30 April 2024, as part of a concurrent workshop titled NRMN-SETH. All data were collected by the first author as a participant-observer. All participants were aware and consented to their slides being collected by the session coordinator. This study was approved as exempt by the Institutional Review Board (IRB) at the first author’s institution.

The session provided an overview of three mentoring models: coaching, developer, and mock study section models, which were designed to equip ESIs with strategic tools for grant-writing and research methodology, centered around rigor and reproducibility, to help refine and articulate their grant proposals. Participants included ESIs from various academic ranks and disciplines, a team of NIH-experienced reviewers involved in grant-writing coaching, a project biostatistician who served as a reviewer in mock study sections, and the program principal investigator.

Presentations by RCMI NRMN-SETH scholars who had received NIH-funded awards highlighted successful grant-writing strategies and shared experiential insights. The session also included interactive discussions among NRMN-SETH scholars, audience members, and RCMI institutional stakeholders. These discussions offered a collaborative platform to address challenges faced by investigators, particularly those developing interinstitutional pilot project proposals or refining applications based on NIH summary statements.

### 2.3. Analysis

Data analysis used directed content analysis [24] to explore the stakeholders, contexts, and mechanisms influencing grant writing and participation among RCMI scholars. The realist evaluation framework informed the context-mechanism-outcome framework for analyzing the NRMN and its impact on biomedical workforce development, focusing on ESIs at minority-serving institutions. This method, guided by the realist evaluation framework, focused on identifying elements critical to understanding “what works, for whom, and under what contexts.” Stakeholder identification, based on mentions in slides and conversations, was integrated to enrich the analysis.

## 3. Results

### 3.1. Summary of Presentations

The session’s presentations and discussions highlighted the value of the NRMN-SETH coaching and mock study section models in supporting ESIs. RCMI scholars shared their research experiences and described their participation in the NRMN-SETH grant-writing coaching program. This program, structured around group meetings every two to three weeks, integrates discussions on grant-writing strategies with peer reviews of proposal drafts. Coaches facilitate these sessions, offering technical feedback while also serving as mentors to support investigators. One coach emphasized the importance of relationship-building and consistent communication in fostering a trusting and psychologically safe environment. This approach, coupled with guidance and accountability, helps investigators remain engaged and productive over the course of six to nine months. Presentations underscored the significance of social aspects, such as network development, as integral components of the NRMN-SETH coaching approach.

Mock study sections emerged as a particularly impactful feature of the program. Though initially optional, they were made a required component in subsequent cohorts due to their demonstrated effectiveness. Preliminary data suggest that participation in mock study sections significantly increases the number of grant proposals submitted. While it is premature to evaluate the success rates of these submissions, the program shows promise as a career and program development model for RCMI ESIs. Future programmatic enhancements should include increasing participation by NIH-funded RCMI investigators, providing access to subject-matter experts, and ensuring protected time for participants to focus on grant-writing activities. These steps are expected to strengthen the model’s effectiveness and scalability.

### 3.2. What Works

Five mechanisms emerged as essential to successful and sustained grant proposal development through the NRMN-SETH coaching model. (1) Social Support: All presenters emphasized the importance of social support as a foundational component. Coaches noted that peer groups helped normalize the challenges of developing grant proposals, fostering an essential support structure. Participants reported that collaborating with peers expanded their subject-matter expertise and their access to additional resources. While unanticipated, many participants formed collaborative relationships, enabling them to obtain substantive feedback and participate in joint project development. (2) Peer Accountability: Regularly scheduled sessions provided participants with a sense of accountability, enabling consistent progress. The structure of these sessions encouraged participants to stay engaged and meet deadlines. (3) Knowledge of Grant-Writing Strategies: Participants reported a significant increase in their understanding of NIH grant mechanisms, such as R and K awards. They learned to develop proposal sections strategically, identify strengths and weaknesses, and tailor their approach based on specific grant requirements. (4) Technical Grant-Writing Knowledge: Participants highlighted the critical technical knowledge gained through the coaching program, including insights into NIH study sections and the roles of scientific review officers and program officers. This understanding facilitated more informed and confident grant submissions. Furthermore, the program’s mock review sessions enabled ESIs to articulate their understanding of the pros and cons of their proposals, thereby enhancing the likelihood of submitting proposals. (5) Access to Mentoring: Both coaches and participants recognized mentoring as a pivotal element of the program. While the program’s initial focus was on the technical aspects of grant writing, it later emphasized mentoring proposal pacing, timeline management, and leveraging pilot projects. This mentoring fostered a supportive environment, enhancing participants’ readiness for sustained grant development.

### 3.3. For Whom

Four key stakeholder groups were identified as essential to the success and sustainability of grant proposal development within the NRMN-SETH coaching model: participants, coaches, peers and collaborators, and organizational leadership. Participants were the primary stakeholders and core recipients of the program. Their diverse backgrounds and varying levels of grant-writing readiness shaped their engagement and progress within the program. Coaches were recognized as critical facilitators, dedicating significant time to providing group and individual feedback, often exceeding the formal scope of their roles. Sixteen senior investigators served as coaches, demonstrating remarkable commitment. However, coaches noted challenges in group composition. Small groups often lacked robust discussions and were vulnerable to delays when participants missed sessions. Conversely, large groups of more than six participants were difficult to manage, leading to extended sessions and limited opportunities for individual feedback. Optimizing group size was highlighted as essential for effective coaching.

Peers and collaborators played a complementary role. While the program primarily focused on individual skill development for primary investigators, participants also contributed to and benefited from the collective group experience. Their participation and co-participation created a two-way learning environment that underscored the importance of mutual support within the group dynamic. Furthermore, the group format of grant writing coaching promoted a sense of accountability, consistency in participation in coaching meetings, and diligence in writing progress. Organizational leaders within the RCMI consortium, particularly core directors of institutional development services, were identified as another crucial stakeholder group. Their recognition and support at the departmental or organizational level were essential to ensuring the long-term success of the RCMI scholars. Examples of scholar backgrounds and outcomes are presented in Table 1.

### 3.4. In What Contexts

Three critical contexts emerged as essential to successful and sustained grant-writing efforts within the NRMN-SETH program. First, formalized protected time for grant-writing activities proved vital for participants’ success. RCMI investigators were asked about their teaching loads and available time upon application. As the program progressed, it became evident that departmental support in providing structured, protected time significantly enhanced participants’ ability to engage with the program effectively and meet grant submission goals. Second, while NRMN-SETH coaches offered technical guidance and accountability, they were not well-positioned to provide specialized subject-matter expertise. Participants who already had established networks of local or collaborative experts, or who proactively sought such expertise, demonstrated greater success in developing proposals that emphasized the significance and innovation of their projects. Access to these experts was a key differentiator in the quality and competitiveness of the proposal. Third, regular participation and consistent engagement with coaching sessions scaffolded successful proposal submissions. Participants who prioritized these activities effectively leveraged the program’s resources, while those who missed sessions faced setbacks in their progress. Institutional support in aligning priorities and providing time flexibility was crucial in fostering participant persistence.

## 4. Discussion

This paper reports on the content and discussions at the NRMN SETH session held at the 2024 RCMI Annual Meeting. The session targeted ESIs affiliated with RCMI institutions while engaging a broader audience, including RCMI mentors, Institutional Development Core directors, and NRMN alumni, scholars, coaches, and developers. Their active participation in discussions enriched the dataset, providing diverse perspectives on the systemic and individual factors influencing grant-writing success. Due to the applied nature of the project, this study did not follow a rigorous qualitative design and analysis protocol. Despite this limitation, the data and findings presented in this paper provide valuable scaffolding for future researchers to better prepare grant-writing training sessions. Through directed content analysis of these multifaceted data sources, this study applied a realist evaluation framework to illuminate the contexts, mechanisms, and outcomes that promote equitable participation in biomedical research funding and sustainable grant writing and submission efforts among ESIs. The results aim to inform strategies for building inclusive research ecosystems that promote transparency, collaboration, and sustainable capacity development among underrepresented investigators.

The findings from this session underscore several critical implications for the future planning and activities of RCMI institutions. These implications center on the importance of investing in mentoring, building networks of subject-matter experts, fostering grant readiness, and expanding research capacity on a broader scale [25]. RCMI institutions are well-placed to utilize their existing networks to find and connect with subject-matter experts and mentors, offering support that goes beyond institutional and organizational limits. Mentors play a vital role in fostering the career development of junior investigators through sustained relationships, while subject-matter experts can offer targeted, short-term consultation to enhance the quality and competitiveness of grant proposals. These efforts require distinct but complementary strategies: cultivating long-term mentoring relationships and building accessible, inter-organizational networks of expertise.

Investing in community sustainment is another key opportunity for RCMI institutions. One recommended strategy is to encourage past participants of programs like NRMN-SETH to become mentors or coaches themselves. Having experienced the benefits of this coaching model, many participants expressed a willingness to pay it forward by supporting their junior colleagues. This approach not only expands the pool of mentors and subject-matter experts but also reinforces a culture of mutual support and collective advancement within the RCMI network. The RCMI Coordinating Center is positioned to facilitate standards and metrics to incentivize research mentoring through RCMI organizational policies and activities [25].

Grant submission readiness is another critical area for investment. Submitting an NIH grant is a complex process that requires substantial preparation, including pilot projects and collaboration with institutional and external partners. RCMI institutions should ensure that investigators have access to protected time for research and grant writing, as well as mentoring tailored to their career stage. Structured support for investigators preparing K- or R-level grants can significantly enhance their readiness and success rates. Cross-institutional collaborations, including joint paper writing and pilot grant applications, offer low-risk opportunities for team development and capacity building.

Strategically, RCMI institutions can move beyond siloed approaches by creating cross-institutional systems that capitalize on existing infrastructure and networks. These strategies should aim to integrate resources across the RCMI consortium and promote collaborative initiatives that align with shared goals. Finally, RCMI institutions face an important decision about their role in supporting investigators at non-RCMI institutions. Research capacity is a shared concern across all institutions, and RCMI investigators are uniquely positioned to serve as a hub for advancing health equity research. By leveraging their internal infrastructure and academic networks, RCMI institutions can extend their impact beyond the consortium, fostering national and global collaboration in impactful biomedical and health research.

## 5. Conclusions

The NRMN-SETH program underscores the critical role of mentorship, community sustainment, participant diligence, and institutional collaboration in advancing biomedical research capacity and health equity. By demonstrating how specific mechanisms, such as peer accountability and technical grantsmanship knowledge, interact with institutional contexts, such as protected writing time, the program provides a reproducible blueprint for building research infrastructure at minority-serving institutions. To move beyond individual success to systemic capacity building, RCMI institutions must transition from temporary interventions to permanent research support ecosystems. This requires sustainable institutional investment in incentivizing mentorship, formalizing grant-readiness pipelines, and fostering cross-institutional networks that provide ESIs with continuous access to subject-matter expertise. In conclusion, research capacity is not merely an individual skill set but a collective institutional asset. A multi-faceted approach that weaves together mentorship, expertise, and intentional policy-driven collaboration will strengthen the RCMI network and amplify its contributions to biomedical and health research, both within and beyond the consortium.

## Figures and Tables

**Table 1:** Examples of NRMN-RCMI Scholar Backgrounds and Outcomes.

Scholar Title at the Time of Participation (e.g., Assistant/Associate Professor)	Discipline	Target NIH Mechanisms (e.g., R01, K99/R00, R15)	Grant Submission Outcome	Other Relevant Career Advancements
Assistant Professor	Cancer Biology	R03, R01, R15, R16	1. R03 awarded 2. R01-type under the U54 grant awarded	Promotion with tenure to Associate Professor
Assistant Professor	Human Nutrition	R21	R21 awarded	Promotion with tenure to Associate Professor
Assistant Professor	Epidemiology	K01	K01	

## Data Availability

Data reported in this publication are available from the author upon request.
